# The Functions of β-Defensin in Flounder (*Paralichthys olivaceus*): Antibiosis, Chemotaxis and Modulation of Phagocytosis

**DOI:** 10.3390/biology10121247

**Published:** 2021-11-29

**Authors:** Xiaokai Hao, Heng Chi, Xiaoqian Tang, Jing Xing, Xiuzhen Sheng, Wenbin Zhan

**Affiliations:** 1 Laboratory of Pathology and Immunology of Aquatic Animals, KLMME, Ocean University of China, 5 Yushan Road, Qingdao 266003, China; hao_xiao0616@163.com (X.H.); tangxq@ouc.edu.cn (X.T.); xingjing@ouc.edu.cn (J.X.); xzsheng@ouc.edu.cn (X.S.); wbzhan@ouc.edu.cn (W.Z.); 2 Laboratory for Marine Fisheries Science and Food Production Processes, Qingdao National Laboratory for Marine Science and Technology, Qingdao 266237, China

**Keywords:** β-defensin, antibiosis, chemotaxis, phagocytosis, extracellular traps

## Abstract

**Simple Summary:**

The study identified a new spliced isoform of anionic β-defensin from flounder (*Paralichthys olivaceus*, fBD) and examined its antibiosis, chemotaxis and modulation of phagocytosis. It also analyzed the contributions of fBD to the antimicrobial activity of extracellular traps (ETs). The analyses found that an anionic β-defensin in fish possesses strong bacteriostatic ability in line with that of cationic defensins and also plays an important role in immune response. This study provides new insights into the biological function of anionic defensins, which can serve as one of the important effectors in extracellular traps and contribute to the immune response.

**Abstract:**

Most defensins are cationic antimicrobial peptides with broad-spectrum killing activity against bacteria, fungi and enveloped viruses. However, it should be recognized that there are some non-cationic β-defensins in organisms, which need to be further studied. In this study, a new spliced isoform of anionic β-defensin from flounder (*Paralichthys olivaceus*, fBD) was identified, and its antibiosis, chemotaxis and modulation of phagocytosis were examined. In addition, the contributions of fBD to the antimicrobial activity of extracellular traps (ETs) were also analyzed. The recombinant fBD (rfBD) could effectively inhibit the growth of Gram-positive bacteria (*S. aureus*, *Micrococcus luteus*) and Gram-negative bacteria (*E. coli*, *V. alginolyticus*, *V. anguillarum*). An indirect immunofluorescence assay showed that the fBD was co-localized in the extracellular traps released by the leukocytes. When the ETs were blocked with antibodies against rfBD, the proliferation of *S. aureus* and *E. coli* incubated with ETs tended to increase compared with that in the control group. In addition, the results obtained by flow cytometry showed that the rfBD could significantly chemoattract leukocytes and increase phagocytic activity in vitro. In conclusion, this study provides new insights into the biological function of anionic defensins, which can serve as one of the important effectors in extracellular traps and as a bridge between innate and adaptive immunity in teleosts.

## 1. Introduction

The immune system of vertebrates is divided into two parts: innate immunity and adaptive immunity. As the first line of defense of the immune system, innate immunity fulfills powerful functions and plays an important role. For lower vertebrates such as bony fish, the innate immune system is particularly important because the formation of an adaptive immune response takes a long time and is affected by the ambient temperature [1]. Antimicrobial peptides, are small-molecule peptides, also known as host defense peptides, usually consisting of 10–60 amino acids, with the characteristics of high temperature resistance, strong cationicity and hydrophobicity [2].

Bony fish, as aquatic temperature-changing vertebrates, are directly exposed to the water environment rich in microorganisms. When the body is damaged or invaded by pathogenic microorganisms, the innate immune system is quickly activated, releasing AMPs, which are important substances to perform its functions of antibiosis. Up to now, fish antimicrobial peptides have been primarily divided into piscidins, defensins, hepcidins, cathelicidins and NK-lysins [3]. Defensins are short, cysteine-rich cationic host defense peptides, divided into α-, β- and θ-defensins in vertebrates [4]. The disulfide bond pairing methods and the positions of the six cysteine residues forming the disulfide bonds of defensins are different [5]. The three disulfide bonds of α-defensin are connected by C1–C6, C2–C4 and C3–C5, while the pairing method of the six cysteines of β-defensin is C1–C5, C2–C4 and C3–C6. In addition, θ-defensin, which also contains three pairs of disulfide bonds, is a cyclic peptide that has only been identified in non-human primates, such as rhesus monkeys [6]. In vertebrates with a low degree of evolution (such as birds and fish), merely β-defensins have been identified, and none of the α-defensins or θ-defensins has been discovered [7]. In fish, zebrafish β-defensin was first identified, and multiple defensin-like genes were subsequently reported in many other species [8,9,10,11,12,13,14].

Most β-defensins are cationic antimicrobial peptides with broad-spectrum killing activity against bacteria, fungi and enveloped viruses [15]. In addition to these antimicrobial activities, β-defensin participates in the interaction between the organism and microorganisms by regulating the phagocytic activity of phagocytes and the chemotaxis of immune cells. As a bridge between adaptive and innate immunity, β-defensin is a potential immune booster candidate for vaccines [16,17]. In teleosts, some studies have demonstrated that cationic β-defensins contributed to the antibiosis [18], inflammatory inhibition [19], leukocytic chemotaxis [20], modulation of phagocytosis and boost of viral DNA vaccines [21].

In higher vertebrates, after being stimulated, several types of immune cells release chromatin and granular proteins into the extracellular space, forming DNA traps [22]. This process occurs in many innate immune cells and is especially prominent in neutrophils. Its components including DNA, histones, elastase, myeloperoxidase, lactoferrin and defensins are involved in the regulation of innate and adaptive immunity [23,24]. However, there was no report about the function and mechanism of defensin in neutrophils or extracellular traps in teleosts.

Flounder (*Paralichthys olivaceus*) is an financially profitable teleost species extensively cultured in East Asian countries [25]. Multiple anionic β-defensin isoforms from flounder have been identified, and the mature peptide’s antimicrobial activities have been found [26]. Nevertheless, as an anionic antimicrobial peptide, it is poorly understood whether the function of β-defensin in flounder resembles that of cationic antimicrobial peptides. In this study, a new anionic β-defensin isoform from flounder (fBD) was identified, and its antibiosis, chemotaxis and modulation of phagocytosis were examined. In addition, the contributions of fBD to the antimicrobial function of extracellular traps were also analyzed.

## 2. Materials and Methods

### 2.1. Experimental Animals

Healthy flounders (*Paralichthys olivaceus*) were purchased from a marine farm in Rizhao city of Shandong Province, China. Then, the flounders were cultured in tanks containing aeration-filtered seawater at 20 ± 0.5 °C for a week before the experiments and fed with dry pellets daily. Mice were obtained from Qingdao Animal Experimental Center of Shandong Province, China, and used for antibody production. This study was executed strictly following the procedures in the Guide for the Use of Experimental Animals of the Ocean University of China in adherence with the International Guiding Principles for Biomedical Research Involving Animals (EU 2010/63). All measures were followed to minimize fish suffering.

### 2.2. RNA Extraction and cDNA Synthesis

The Trizol method was used to extract total RNA from head kidney of flounder [27]. RNA was eluted in 30–50 µL nuclease-free water and quantified using the Nanodrop ND-8000 spectrophotometer (Thermo, Waltham, MA, USA). The synthesis of cDNA was performed with SuperScript II Reverse Transcriptase (Vazyme, Nanjing, China) according to the instruction provided by the producer.

### 2.3. Cloning and Sequence Analysis

Nested PCR was used to amplify the cDNA of fBD with outer primers fBD-OF/fBD-OR and inner primers fBD-IF/fBD-IR (Table 1) according to the sequences deposited in GenBank (accession No. GQ414992). The PCR products were purified using a DNA gel extraction kit (Axygen, Hangzhou, China) and cloned into a pET-32a vector. The plasmid containing PCR products was then transformed into *E. coli* (DE3, Tsingke, Qingdao, China). Plasmids from five individual clones were purified using the EasyPure Plasmid MiniPrep kit (TransGen Biotech, Beijing, China) and sequenced using T7F and T7R primers (Table 1).

The BLAST program, ExPASy Molecular Biology Server (http://us.expasy.org (accessed on 21 August 2021)) and Pfamp were used to analyze the cDNA sequences and to deduce the amino acid sequences of the fBD. A phylogenetic tree was structured using the maximum-likelihood method according to nucleotide sequences by using MEGAX.

### 2.4. Preparation of Recombinant fBD

The positive clone was cultured in 500 mL of LB medium containing 50 μg mL^−1^ ampicillin at 37 °C until the OD_600_ reached 0.6 (~2 × 10^9^ CFU mL^−1^). The isopropyl b-D-thiogalacto pyranoside was used as an induction agent. Affinity purification of recombinant fBD–Trx fusion protein was performed using His Trap™ HP Ni-Agarose (GE Healthcare China, Beijing, China) based on the instructions. Protein concentration was adjusted to 1 mg mL^−1^ by using the BCA Protein Assay Kit (Solarbio, Beijing, China). Subsequently, 10 U of enterokinase (Yaxinbio, Shanghai, China) was used to cleave Trx tags at 4 °C for 24 h. The recombinant fBD (rfBD) was separated through the use of His Trap™ HP Ni-Agarose (GE Healthcare China) by collecting the flow-through containing cleaved protein. The products of each process were verified by sodium dodecyl sulfate polyacrylamide gel electrophoresis (SDS-PAGE).

### 2.5. Antimicrobial Activity Assay

The minimum inhibitory concentrations (MICs) of rfBD for two Gram-positive bacteria (*S. aureus* and *M. luteus*) and three Gram-negative bacteria (*E. coli*, *Vibrio lysis* and *V. anguillarum*) was determined using a dilution method according to a previous report [28]. The strains were isolated from the flounder and proved in our laboratory [29,30]. In brief, twofold serial dilutions of rfBD in sterilized MH medium (Land, Beijing, China) were produced in sterile 96-well microtiter plates with each well loaded with 100 μL of the diluted solution except for the last well. The bacteria (OD_600_ = 0.5, ~1 × 10^9^ CFU mL^−1^) were diluted 100-fold in MH medium, and then 100 μL of liquid was added into each well except for the first well. The first and last wells were given 100 μL MH medium without bacteria as the control group. The 96-well microtiter plates (Corning, New York, NY, USA) were incubated at 30 °C for 24 h, and the OD_600_ values of the plates were then recorded by using a microplate reader (Tecan, Canton of Zurich, Switzerland). The MIC values were noted as a range between the highest concentration of the protein at which bacterial growth was observed and the lowest concentration that resulted in 100% inhibition of bacterial growth.

### 2.6. Production of Antibody and Indirect Immunofluorescence Assay

The production of polyclonal antibody against rfBD was performed as previously indicated [31]. The BALB/c mice were immunized four times at biweekly intervals with 20 mg purified rfBD protein. Following four boosts, the serum samples were obtained and anti-rfBD polyclonal antibodies were purified using protein G agarose affinity chromatography (Thermo). The sterile PBS was injected into the abdominal cavity of the flounder and then withdrawn with a syringe. After being washed with PBS, 20 μL of peritoneal cells (5 × 10^6^ cells mL^−1^) was dropped and settled on APES-coated slides for 1 h. The cells were fixed by 4% formaldehyde (Thermo) for half an hour. After incubation with 5% BSA for 2 h at room temperature, the peritoneal cells were treated with anti-rfBD for 1 h. After washing three times with PBS, the peritoneal cells were treated with Alexa Fluor 488–conjugated goat anti-mice IgG (1:1000, Sigma, Nawa Prefecture, Japan) for 45 min at 37 °C in the dark. After washing three times with PBS, the peritoneal cells were counter stained with 4,6-diamidino-2-phenylindole (DAPI, Bio-Legend, Santiago, Chile) and then viewed using a Zeiss fluorescence microscope (Zeiss, Jena, Germany).

### 2.7. Co-localization rfBD and Extracellular Traps

The co-localization study was performed using an immunofluorescence method in line with previously studies [32]. In brief, the leukocytes were isolated from the head kidney using a discontinuous Percoll (GE Healthcare, Chicago, IL, USA) gradient (1.02/1.07) and seeded onto glass coverslips that had been soaked with polylysine (Sigma, St. Louis, MI, USA); they were then placed in a 12-well cell culture plate (Corning). The 12-well cell culture plate was settled for 2 h in L15 medium (HyClone, Logan, UT, USA), and then the PMA (500 ng mL^−1^, Sigma) was added at 22 °C for 3 h to induce extracellular trap (ET) release. The leukocytes and ETs were fixed with 2% formaldehyde (Sigma) for 30 min and then cleaned with PBS. The coverslips were treated with 5% BSA for 2 h and subsequently treated with antibodies against rfBD at 37 °C for 1 h. After washing with PBS, the coverslips were incubated with Alexa Fluor 488–labeled goat anti-mouse IgG (1:2000, Thermo) at 37 °C for 1 h. After staining with DAPI (Bio-Legend), they were then viewed by a fluorescence microscope (Zeiss).

### 2.8. Survival of ETs-Trapped Bacteria

Bacteria [*E. coli, Edwardsiella tarda* (*Ed. tarda*) and *S. aureus*] were cultured in LB medium until OD_600_ was 0.5 (1 × 10^9^ CFUs mL^−1^) and enriched in L15 medium. Leukocytes (2 × 10^5^ cells well^−1^) from the head kidney were distributed in 96-well cell culture plates (Corning) and allowed to settle for 60 min at room temperature. The leukocytes were treated with 500 ng mL^−1^ PMA for 2 h to induce extracellular trap release, and the ET-negative group was not stimulated with PMA. Subsequently, 20 μg mL^−1^ of cytochalasin D (Invitrogen, Carlsbad, CA, USA) and anti-fBD (1:1000) were added to the wells. After incubation for 2 h, 2000 CFUs of bacteria in 100 μL of L15 medium were added to the corresponding wells. After incubation for 2, 4 or 8 h, the solution from each well was diluted and spread on bacterial culture plates. After incubation for 12 h, the colonies that appeared on the plates were then recorded according to a reported previously method [32].

### 2.9. In Vitro Chemotaxis Assay

The chemotactic activity of the rfBD was evaluated by attracting the leukocytes of flounder from the head kidney and spleen using Transwell Permeable Supports (Corning) [19,28]. According to the diameter of the cells, transwells of 8 μm pore size were selected. The chemotaxis of the rfBD on leukocytes was evaluated in a 24-well plate. This experiment was carried out with rfBD (2 μg mL^−1^, using sterile L15 cell culture medium) or with rTrx as a control. Briefly, 600 μL of rfBD or rTrx dilution in L15 medium were added to the lower chamber of the transwell, and 100 μL of leukocytes (10^5^ cells) were placed in the chamber of the insert. After 4 h of incubation at room temperature, flow cytometry (20 μL of solution from lower chamber, Accuri C6, BD, NJ, USA) was used to estimate the number of leukocytes that migrated into the lower chamber.

### 2.10. Phagocytic Assay

According to the method described previously [27,33], the leukocytes were suspended in serum-free L-15 medium (10^7^ cells mL^−1^) and transferred to 24-well culture plates (500 μL well^−1^, Corning). The leukocytes were then incubated with rfBD (2 μg mL^−1^) or with rTrx (2 μg mL^−1^) for 4 h, green-fluorescent microspheres were added into the wells at the ratio to cells of 20:1. After incubation for 3 h, the leukocytes were collected and cleaned with PBS and analyzed with a flow cytometer (Accuri C6, BD).

### 2.11. Statistical Analysis

Statistical Products and Services Solutions (SPSS) was used to conduct statistical analyses. Independent-sample t-test and Duncan’s multiple polar difference test (DMRT) were used to analyze statistical significance. Data were expressed as mean ± standard deviation, and the difference was considered significant at *p* < 0.05 and highly significant at *p* < 0.01.

## 3. Result

### 3.1. Cloning and Sequence Analysis of fBD

**Figure 1:**
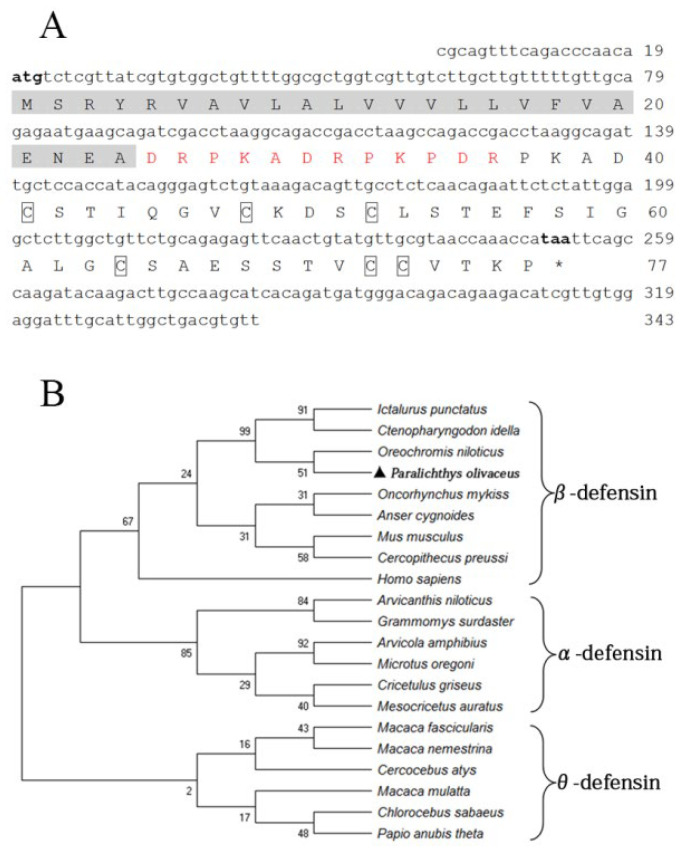
Sequence structure and phylogenetic analysis of fBD. (**A**) Nucleotide and amino acid sequences of fBD. The initiation and termination codons are marked with bold letters. The signal peptide is marked with a highlight, the propiece region is shown in red, and fixed cysteines are framed by boxes. (**B**) Phylogenetic tree analysis of rfBD from flounder and other species. The tree was structured according to the “maximum-likelihood” method using MEGAX. The GenBank accession numbers of α-, β- and θ-defensins are listed as follows: *Homo sapiens*: AF529417.1; *Ctenopharyngodon idella*: MG652749.1; *Oncorhynchus mykiss*: NM_001195183.2; *Anser cygnoides*: EU606039.1; *Mus musculus*: AY591385.1; *Oreochromis niloticus*: MH674361.1; *Ictalurus punctatus*: KX211992.1; *Cercopithecus preussi*: EU126877.1; *Arvicanthis niloticus*: XM_034520497.1; *Arvicola amphibius*: XM_038325539.1; *Cricetulus griseus*: XM_027435861.1; *Grammomys surdaster*: XM_028784038.1; *Mesocricetus auratus*: XM_040733512.1; *Microtus oregoni*: XM_041673564.1; *Cercocebus atys*: XM_012078007.1; *Chlorocebus sabaeus*: XM_037984733.1; *Macaca fascicularis*: XM_005562541.2; *Macaca mulatta*: AF191100.1; *Macaca nemestrina*: XM_011711827.2; *Papio anubis*: FJ030939.1; *Paralichthys olivaceus*: OL631146.

The fBD (GenBank accession No. OL631146) contained a 234 bp open reading frame (ORF) encoding 77 amino acids with a molecular weight of 13.25 kDa and a theoretical isoelectric point (pI) of 4.8. Six conserved cysteines were present in the amino acid sequence. The protein comprises a signal peptide (24 amino acids) with a pI of 4.0 and a mature peptide (53 amino acids) with a pI of 5.7, in theory (Figure 1A). The phylogenetic relationship of fBD to other species’ β-defensin genes was examined through an analysis of the ORF sequence. The fBD genes clustered with those of the Nile tilapia at a branch and clustered with those of channel catfish and grass carp at a high level of statistical confidence (Figure 1B).

### 3.2. rfBD Production and Antibody Verification

**Figure 2:**
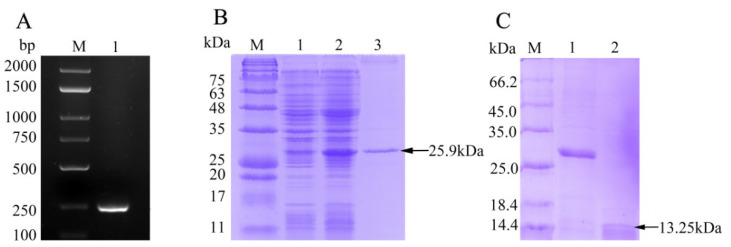
The preparation of recombinant fBD. (**A**) The agarose gel electrophoresis of fBD gene amplified from head kidney by PCR. Lane M, DNA marker; lane 1, PCR products of fBD. (**B**) SDS-PAGE analysis of rfBD peptide. Lane M, protein marker; lane 1, uninduced whole-bacteria lysate; lane 2, induced whole-bacteria lysate; lane 3, purification of rfBD peptide with Trx tag. (**C**) SDS-PAGE analysis of rfBD peptide after digestion. Lane M, protein marker; lane 1, purification of rfBD peptide with Trx tag; lane 2, purification of rfBD peptide without Trx tag. Full figure of PAGEs can be found in Supplementary Materials.

**Figure 3:**
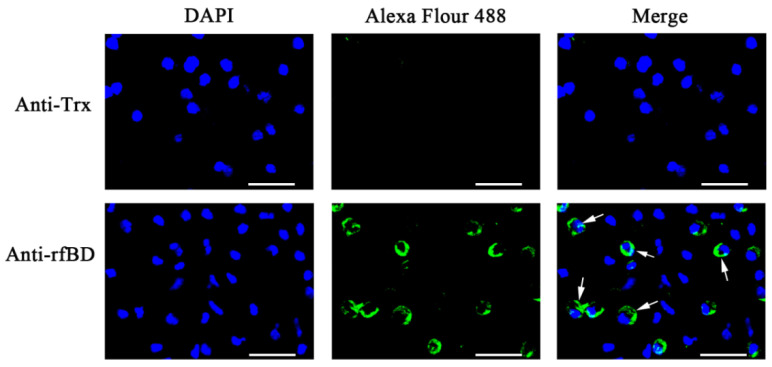
The localization of fBD in peritoneal cells. After incubation with anti-rfBD or anti-Trx antibody, the peritoneal cells were detected using Alexa Flour 488–labeled secondary antibody. The nucleus was visualized using DAPI. In both cases, the right panels were merges of the left and middle panels. The arrows indicate some representative fBD-positive cells. Bar, 20 μm.

The ORF of fBD gene was cloned via PCR from the head kidney transcriptome (Figure 2A). The PCR product was successfully inserted into the vector pET-32a and verified using gene sequencing. rfBD fused with Trx tag was successfully induced and purified. The purified rfBD–Trx fusion protein was verified by SDS-PAGE (Figure 2B, theoretical size approximately 25.9 kDa). The Trx tag of the rfBD was successfully cleaved, and the rfBD without Trx tag was purified (Figure 2C, 13.25 kDa). Peritoneal cells of flounder were used to verify the specificity of the antibodies via immunofluorescence microscopy. As shown in Figure 3, the prepared antibody can be perfectly combined with the natural fBD protein in the cytoplasm.

### 3.3. Antimicrobial Activity of rfBD

**Figure 4:**
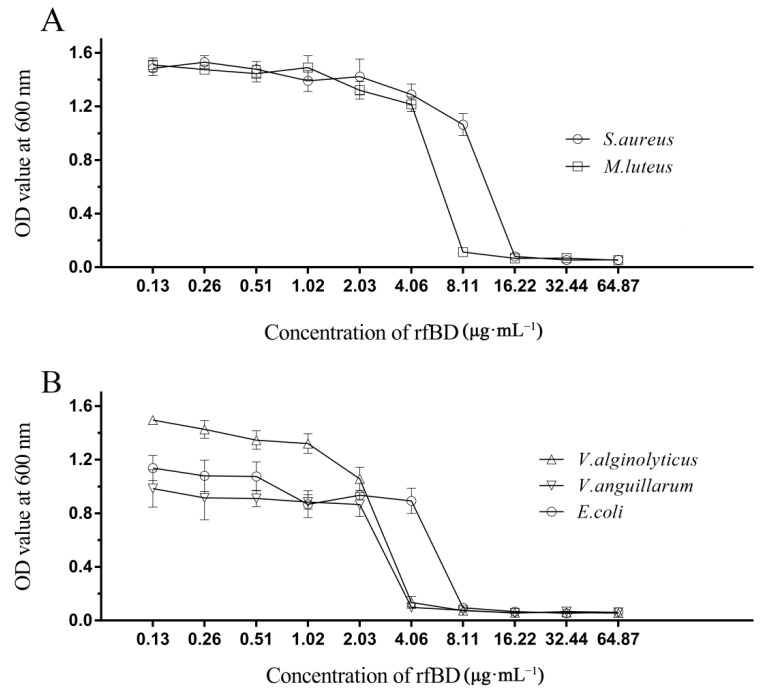
Antibacterial activities of rfBD. The broth dilution method was used to study the inhibitory effects of different concentrations of rfBD on two Gram-positive bacteria (**A**) and three Gram-negative bacteria (**B**). The results were expressed as the absorbance value of the culture solution at 600 nm (The data was summarized in Table S1). The values in the graph are expressed as mean ± SD (*n* = 3).

The dilution method was used to establish the MICs of rfBD against bacteria. rfBD showed significant concentration-dependent growth-inhibitory activity against *S. aureus*, *M. luteus*, *E. coli*, *V. alginolyticus* and *V. anguillarum* (Figure 4, Table 2). The MIC values are summarized in Table 2. 

### 3.4. Localization and Effect of fBD on the ETs

**Figure 5:**
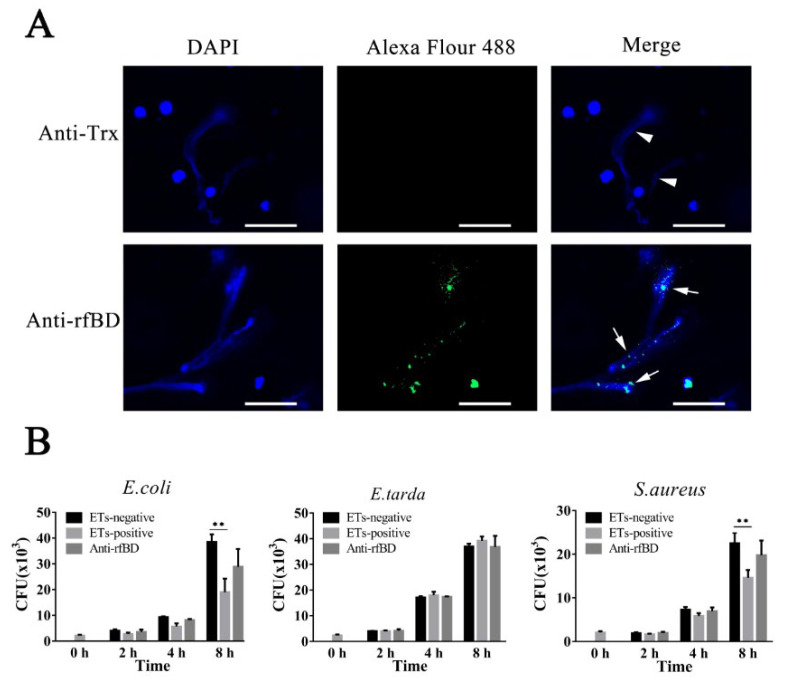
The localization and antibacterial activities of fBD in extracellular traps. (**A**) PMA-stimulated ET-producing cells were treated with antibodies against Trx or rfBD followed by Alexa Fluor–labeled secondary antibody. Arrows without tail indicate ETs and arrows with tails indicate fBD in ETs. Bar, 20 μm. (**B**) Effect of rfBD on bacteriostatic capabilities of ETs. ET-producing cells were infected by *E. coli*, *Ed. tarda* and *S. aureus* with or without (control) of anti-rfBD, and bacterial survival was counted at different times. Data are the means of three independent assays and are presented as means ± SEM. ** *p* < 0.01.

To examine whether the fBD was present in ETs, leukocytes of flounder were treated with the ET inducer PMA, and the resulting ETs were verified by immunofluorescence microscopy. As illustrated in Figure 5A, fBDs were detected by antibodies against rfBD co-localized with extracellular DNA fiber. Following the above results, we evaluated the functions of fBDs in conferring the antimicrobial activity of ETs. For this purpose, ET-positive cells were treated with or without antibodies against rfBD before being subjected to *E. coli*, *Ed. tarda* and *S. aureus* infection, and bacterial survival on ETs was monitored at 2, 4 and 8 h after infection. In *E. coli* and *S. aureus* infection groups, the recovery numbers of bacteria from ET-positive cells groups were significantly lower at 4 and 8 h compared to the ET-negative cells. The recovery rate of bacteria from ET-positive cells in the presence of antibodies against rfBD was increased compared to the ET-positive cells that were not treated, at 4 and 8 h after infection; however, there was no significant difference. In comparison, the number of *Ed. tarda*, collected from ET-positive cells with or without anti-rfBD antibodies were similar to those from ET-negative cells at all time points (Figure 5B).

### 3.5. Effects of rfBD on Chemotaxis of Leukocytes

**Figure 6:**
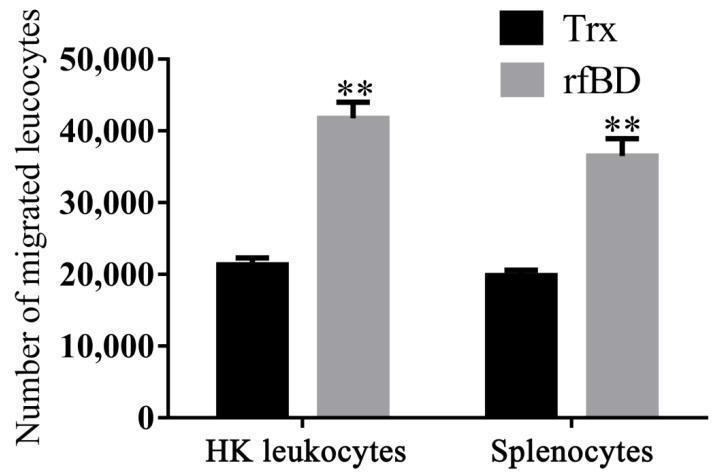
Chemotactic effects of rfBD on leukocytes from head kidney and spleen in vitro. Migrated leukocytes to the lower chamber of the transwell were counted by flow cytometry. Bars represent the mean number of migrated leukocytes ± SEM (*n* = 3). The level of significance compared with the Trx treatment group is represented by ** *p* < 0.01.

By using a transwell, rfBD was proved to have chemotactic ability to leucocytes from the head kidney and spleen. Flow cytometric results revealed that the number of leukocyte chemotaxes to the lower chamber was significantly increased in the presence of rfBD (Figure 6). The chemotactic number of leucocytes from the head kidney was 41,790 ± 2221 cells in the presence of rfBD, while in the control group, it was only 21,350 ± 968 cells. The chemotactic number of leucocytes from the spleen was 36,510 ± 2421 cells in the presence of rfBD, while in the control group, it was only 19,870 ± 728 cells.

### 3.6. Effects of rfBD on the Phagocytosis of Lymphocytes

**Figure 7:**
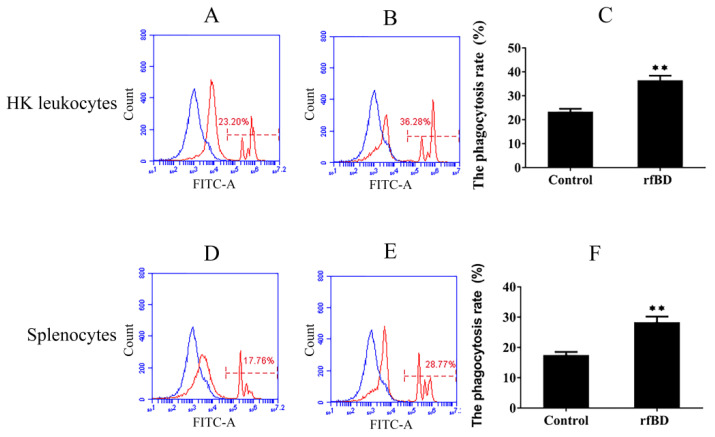
The phagocytosis of leukocytes from head kidney and spleen with or without rfBD stimulation detected by flow cytometric analysis. FITC fluorescence histograms showed the ratio of leukocyte with (**B**,**E**) or without (**A**,**D**) rfBD stimulation. The phagocytosis rate of leukocytes from the head kidney (**C**) and the spleen (**F**), respectively, with rfBD stimulation was analyzed using SPSS. All the error bars represent the standard deviation of three biological replicates. ** *p* < 0.01.

Phagocytosis assay was conducted to investigate whether rfBD can promote the phagocytic capability of leucocytes from the head kidney and spleen. Flow cytometric analysis revealed that leucocytes acquired significantly higher green-fluorescent-microsphere phagocytic activity after rfBD treatment (Figure 7). The phagocytic rate of leucocytes from the head kidney was 36.43% ± 2.02% in the group with rfBD treatment, while in the control group, it was only 23.31% ± 1.29%. The phagocytic rate of leucocytes from the spleen was 28.31% ± 1.90% in the group with rfBD treatment, while that in the control group was only 17.47% ± 1.09%.

## 4. Discussion

Most β-defensin genes are composed of two exons separated by an intron of variable length. However, β-defensin genes in some species contain an additional one or two exons encoding an internal pro-sequence, a segment of carboxyterminal mature sequences or untranslated regions. Alternatively, spliced isoforms have also been discovered with several β-defensins. β-defensin and its cloned isoforms of the flounder from the early developmental stages have been reported [26]. A new spliced isoform of β-defensin from flounder was cloned in this study, encoding peptides with six cysteine residues in a conserved sequence positions that are typical structural characteristics of vertebrate β-defensins. Although most defensins are referred to as cationic antimicrobial peptides, non-cationic defensins have been reported recently. The predicted pI values of β-defensins in *Bombyx mori* and *Amblyomma hebraeum* are 4.44 and 4.12, respectively [34,35]. The new splice isoform of β-defensin consists of a signal peptide with a theoretical pI of 4.0 (24 amino acids) and a mature peptide with the pI of 5.7 (53 amino acids), which is similar to the previously reported pI value of its cloned isomer. Although the predicted pI values of these defensins are different from those of typical defensins, they did not affect the bactericidal functions. According to phylogenetic analysis, fBD was highly similar to defensins from Nile tilapia, grass carp and channel catfish, and it belongs to the group of β-defensins.

Defensins generally possess antibacterial abilities against a broad spectrum of bacteria. The defensin from manila clam showed relatively good antibacterial functions against *Vibrio* [28]; the trx-Defensin from cod has obvious antibacterial functions against *Aeromonas hydrophila* and the recombinant Defb displayed antibacterial activity against *Planococcus citreus* and *Micrococcus luteus*, respectively [33]. In this study, the antimicrobial function of rfBD against bacteria was evaluated. The purified rfBD–Trx fusion protein did not exhibit significant antibacterial activity. When the Trx tag carried by the rfBD was cleaved, its antibacterial activity resumed. rfBD peptides have displayed antibacterial activity against *S. aureus*, *M. luteus*, *E. coli*, *V. alginolyticus* and *V. anguillarum* similar to other defensins [36,37]. Strikingly, we found higher antimicrobial activity of rfBD for Gram-negative bacteria, similar to other antibacterial peptides such as mytichitin-A and gallerimycin from invertebrates [36,37].

ETs had been described both in vertebrate and invertebrate, and their components mainly include DNA, histones, elastase, myeloperoxidase and lactoferrin, which modulate the immune response [23,24,38,39]. In fish, it was found that CsH2B, CsH4, CsEla1 and CsEla2 were localized in ETs and played a role in the antibacterial activity [32]. In this study, we aimed to find out whether fBD, which has strong antibacterial activity, was present on ETs and exerted its antibacterial function. After the ETs were blocked by antibodies against rfBD, *S. aureus* and *E. coli* proliferation tended to increase in comparison with that in the control group, yet no significant variation was caused by experimental conditions. This was for the first time revealed that fBD was located in the extracellular traps in teleosts and contributed to the bacterial inhibition effect.

In mammals, β-defensins are presented at higher concentrations in inflammatory sites and have structures similar to chemokines, suggesting that they might be similar in function [40]. Admittedly, studies have revealed that some overlaps in function between chemokines and defensins in several mammalian and fish species [41,42], such as in seabream, where head kidney leucocytes exhibited chemotactic activity toward supernatants containing β-defensin [20], or in *Megalobrama amblycephala*, where β-defensin can recruit leukocytes. In our study, the rfBD could significantly chemoattract leukocytes from the spleen and head kidney in vitro. In addition, the chemotactic capacity of rfBD for leukocytes from head kidney was higher than that of the leukocytes from the spleen. The reasoning from these results is that there are more phagocytes such as neutrophils and macrophages in head kidney involved in innate immunity, which are easily chemotactic due to chemokines. Some previous studies have demonstrated that α-defensins are known stimulants to modulate phagocytosis [16], In fish, cod Defb increased the phagocytic activity of head kidney leucocytes by over 60%; Manila clam rRpdef1α significantly enhanced the phagocytosis of hemocytes [28]. Notably, rfBD significantly increased phagocytic activity of leukocytes from head kidney and spleen. These results indicated that defensins can act as a bridge between innate and adaptive immunity by chemotaxizing leukocytes and modulating phagocytosis.

## 5. Conclusions

β-Defensin in flounder is an anionic antimicrobial peptide that is localized in the cytoplasm of activated peritoneal cells and on extracellular traps. rfBD could effectively inhibit the growth of Gram-negative and Gram-positive bacteria and could significantly chemoattract leukocytes from the spleen and head kidney in vitro. Moreover, the phagocytosis ability of leukocytes from the head kidney and spleen could be significantly enhanced after incubation with rfBD. The study provides new insights concerning the biological function of anionic defensins on the antibiosis, chemotaxis and modulation of phagocytosis and served as one of the important effect factors in extracellular traps in teleost.

## Figures and Tables

**Table 1:** The name and sequence of primers.

Primer Name	Sequence (5′→3′)
fBD-OF	CGCAGTTTCAGACCCAACAATG
fBD-OR	AAGACGTCAGCCAATGCAAATC
fBD-IF	CGGGATCCATGTCTCGTTATCGTGTGGCT
fBD-IR	CCCTCGAGTTATGGTTTGGTTACGCAACATA
T7F	TAATACGACTCACTATAGGG
T7R	TGCTAGTTATTGCTCAGCGG

**Table 2:** Antimicrobial activities of rfBD determined by liquid growth inhibition assay. * The MIC values were noted as a scope among the highest concentration of the protein at which bacterial growth was observed and the lowest concentration that resulted in 100% inhibition of bacterial growth.

Microorganisms	MIC Value * (μg·mL^−1^)
Gram-positive bacteria	
*S. aureus*	8.11–16.22
*M. luteus*	4.06–8.11
Gram-negative bacteria	
*E. coli*	4.06–8.11
*V. alginolyticus*	2.03–4.06
*V. anguillarum*	2.03–4.06

## Data Availability

The data in this research are accessible upon demand from the corresponding author. E-mail: chiheng@ouc.edu.cn.

## Supplementary Materials

The following are available online at https://www.mdpi.com/article/10.3390/biology10121247/s1, Table S1: The different concentrations of rfBD on antibacterial activity of *S. aureus*, *M. luteus*, *E. coli*, *V. alginolyticus* and *V. anguillarum*. Data represent mean ± S.D. (*n* = 3).
