# Supplementary material for: The Functions of β-Defensin in Flounder (Paralichthys olivaceus): Antibiosis, Chemotaxis and Modulation of Phagocytosis
Source: Biology (Basel). 2021 Nov 29;10(12):1247. doi: 10.3390/biology10121247 (PMC8698591; doi:10.3390/biology10121247)

1. The agarose gel electrophoresis of fBD gene amplified from head kidney by PCR (the full picture of Fig. 2A).

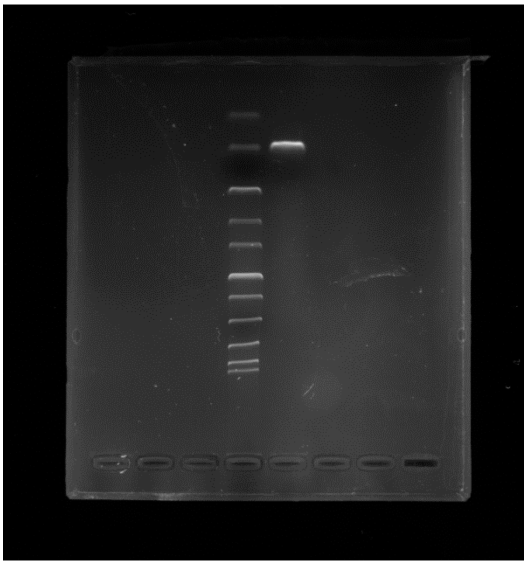

2. SDS-PAGE analysis of rfBD peptide (the full picture of Fig. 2B).

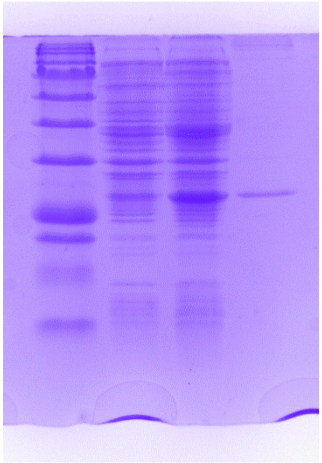

3. SDS-PAGE analysis of rfBD peptide after digestion (the full picture of Fig. 2C).

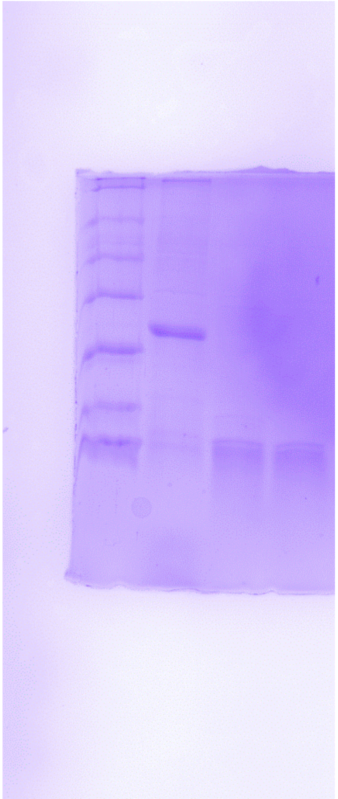

Supplement: Supplementary file 1 [file biology-10-01247-s001.zip › Full figure of PAGEs.pdf]
