# Supplementary material for: The Functions of β-Defensin in Flounder (Paralichthys olivaceus): Antibiosis, Chemotaxis and Modulation of Phagocytosis
Source: Biology (Basel). 2021 Nov 29;10(12):1247. doi: 10.3390/biology10121247 (PMC8698591; doi:10.3390/biology10121247)
Supplement: Supplementary file 1 [file biology-10-01247-s001.zip › Table S1-layout.pdf]

Table S1. The different concentrations of rfBD on antibacterial activity of *S. aureus, M. luteus, E. coli, V. al-ginolyticus* and *V. anguillarum*. Data represent mean ± S.D. (n = 3).

**Concentration ($\mu g \cdot ml^{-1}$)**	**OD value at 600 nm**				
	S.aureus	***M.luteus***	***E.coli***	***V.alginolyticus***	***V.anguillarum***
64.87	0.0556 ±0.009	0.0537 ±0.0006	0.0635 ±0.0107	0.0563 ±0.0111	0.0614 ±0.0113
32.44	0.0539 ±0.012	0.069 ±0.0057	0.0547 ±0.0073	0.0596 ±0.0107	0.0666 ±0.0161
16.22	0.0808 ±0.024	0.0675 ±0.0164	0.0676 ±0.001	0.0584 ±0.015	0.0555 ±0.0083
8.11	1.0653 ±0.0814	0.1135 ±0.0271	0.0952 ±0.007	0.0738 ±0.0204	0.0771 ±0.0074
4.06	1.2897 ±0.0774	1.2165 ±0.0546	0.8934 ±0.094	0.1359 ±0.0452	0.0981 ±0.0178
2.03	1.4225 ±0.1323	1.3207 ±0.0654	0.9355 ±0.034	1.0575 ±0.0867	0.8667 ±0.0891
1.02	1.3913 ±0.0792	1.4905 ±0.0887	0.8681 ±0.1001	1.3206 ±0.0734	0.8854 ±0.0537
0.51	1.478 ±0.0586	1.4453 ±0.0615	1.0766 ±0.1087	1.3468 ±0.0685	0.9114 ±0.0603
0.26	1.531 ±0.0474	1.4754 ±0.0223	1.0805 ±0.1158	1.4266 ±0.0657	0.9155 ±0.1629
0.13	1.486 ±0.0548	1.5108 ±0.0507	1.1389 ±0.0939	1.4966 ±0.0345	0.9854 ±0.1395
0.00	1.5243 ±0.0499	1.5054 ±0.0368	1.2022 ±0.0256	1.485 ±0.0605	1.0657 ±0.0746

	OD value at 600 nm
MH medium	0.0557±0.0085
